# Acetyl-L-carnitine ameliorates atherosclerosis in LDLR^−/−^ mice by modulating cholesterol metabolism through SREBP2-dependent cholesterol biosynthesis

**DOI:** 10.3389/fnut.2024.1509577

**Published:** 2024-12-16

**Authors:** Jingci Xing, Zhiyong Du, Fan Li, Yu Wang, Zihan Zhang, Xiaoqian Gao, Lijie Han, Xuechun Sun, Haili Sun, Yunhui Du, Chaowei Hu, Huahui Yu, Yanwen Qin

**Affiliations:** ^1^Beijing Anzhen Hospital, Beijing Institute of Heart Lung and Blood Vessel Disease, Capital Medical University, Beijing, China; ^2^School of Basic Medical Sciences, Binzhou Medical University, Yantai, China

**Keywords:** atheroscelrosis, cholesterol metabolism, acetyl-L-carnitine (ALC), SREBP2, HMGCR

## Abstract

**Background:**

Atherosclerotic cardiovascular disease (ASCVD) is the leading cause of mortality globally. Hypercholesterolemia accelerates atherosclerotic development and is an independent modifiable risk factor for ASCVD. Reducing cholesterol levels is effective in preventing ASCVD. Acetyl-L-carnitine (ALC) is an endogenous molecule that plays a primary role in energy metabolism; however, its effect on cholesterol metabolism remains unclear.

**Methods:**

We collected plasma samples and clinical data from 494 individuals with hyperlipidemia. Targeted metabolomics were used to measure plasma ALC levels and explore the association of ALC with clinical cholesterol levels. Additionally, we explored the effects of ALC in cholesterol levels and cholesterol metabolism in a murine hypercholesterolemia model. An LDLR^−/−^ mouse-based atherosclerotic model was established to investigate the roles of ALC on atherosclerotic progression.

**Results:**

Plasma ALC concentrations were significantly negatively correlated with plasma total cholesterol (TC) levels (*r* = −0.43, *p* < 0.0001) and low-density lipoprotein cholesterol (LDL-C; *r* = −0.53, *p* < 0.0001). Incorporating ALC into the diet significantly reduced plasma TC and LDL-C levels, downregulated genes involved in cholesterol synthesis, such as sterol regulatory element-binding protein 2 (SREBP2) and 3-hydroxy-3-methyl-glutaryl-CoA reductase, and upregulated low-density lipoprotein receptor expression. ALC supplementation substantially lowered plasma TC levels and inhibited atherosclerosis in LDLR^−/−^ mice.

**Conclusion:**

ALC reduced atherosclerotic plaque formation by lowering plasma cholesterol levels via suppression of SREBP2-mediated cholesterol synthesis, thus suggesting that ALC is a potential therapeutic target for ASCVD.

## Graphical abstract

ALC reduces plasma TC by decreasing SREBP2-dependent cholesterol synthesis, thereby slowing atherosclerotic progression. ALC reduces SREBP2 synthesis, leading to decreased nSREBP2 levels, resulting in reduced HMGCR transcription, lowering cellular HMGCR levels and subsequently decreasing de novo cholesterol synthesis. The reduced plasma TC ultimately slows the atherosclerotic progression. Created from https://Biogdp.com, licensed under CC BY-NC 4.0 (34).

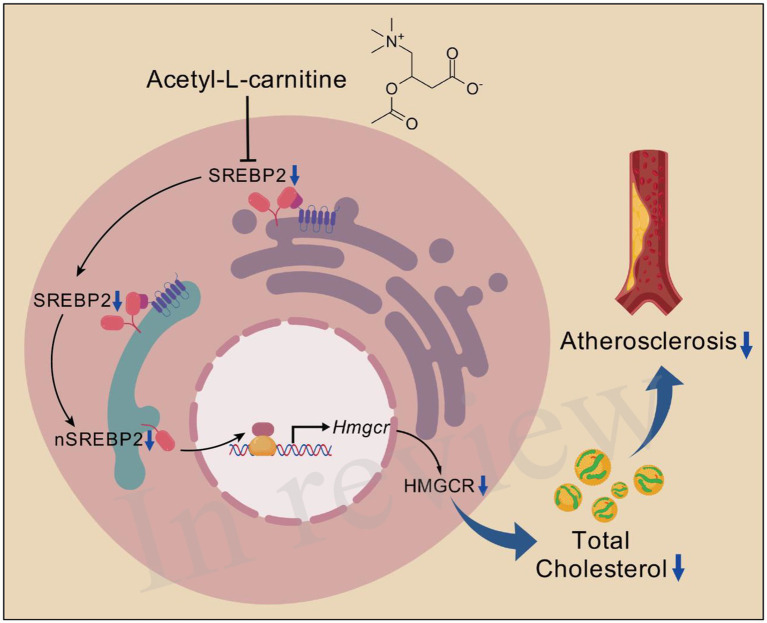

## Introduction

Atherosclerotic cardiovascular disease (ASCVD) is the leading cause of death worldwide (1). Low-density lipoprotein cholesterol (LDL-C) is a major causal factor in the pathophysiology of ASCVD (2). Lowering LDL-C is the primary strategy for preventing and treating atherosclerosis because it significantly reduces cardiovascular morbidity and mortality (3, 4). Statins are the foundation of lipid-lowering therapy, but many patients fail to achieve target LDL-C levels and remain at considerable risk despite treatment (5, 6). This residual risk has led researchers to explore secondary targets, such as non-high-density lipoprotein cholesterol and high-sensitivity C-reactive protein (3). Newer approaches to further lower LDL-C include the inhibition of ATP-citrate lyase, proprotein convertase subtilisin/kexin type 9, angiopoietin-related protein 3, and cholesteryl ester transfer protein (6). Combination therapy with other agents is often necessary to achieve optimal LDL-C reduction in high-risk patients (3). Although these medications lower LDL-C through different mechanisms, at least 20% of patients experience recurrent myocardial infarction within 3 years of their initial clinical presentation, regardless of how well their cholesterol is controlled (7). Therefore, alternative therapies are urgently needed to reduce the morbidity and mortality associated with atherosclerosis.

Acetyl-L-carnitine (ALC) is an endogenous molecule synthesized via the enzyme carnitine acetyltransferase in the human brain, liver, and kidneys (8). ALC plays a crucial role in cellular energy metabolism by facilitating the transport of acetyl groups into the mitochondria during fatty acid oxidation (9). Clinically, ALC has been used as a dietary supplement and therapeutic agent for conditions such as peripheral neuropathy and cognitive decline. Emerging evidence suggests that carnitine derivatives may influence lipid metabolism and cardiovascular health (10, 11). However, the specific effects and mechanisms of ALC on cholesterol metabolism and atherosclerosis remain largely unexplored.

In this study, we investigated the potential role of ALC in cholesterol metabolism and atherosclerosis. We hypothesized that ALC supplementation could modulate cholesterol levels and attenuate atherosclerotic lesion development by influencing the SREBP2 pathway. To test this hypothesis, we studied the effects of ALC on plasma cholesterol levels, atherosclerotic progression, and the expression of genes involved in cholesterol homeostasis. Our findings provide novel insights into the therapeutic potential of ALC for managing hypercholesterolemia and atherosclerosis.

## Materials and methods

### Participants

Between January 2018 and December 2018, 494 patients with or without hypercholesterolemia were enrolled at Anzhen Hospital, Beijing, China. Inclusion criteria were age >18 years, diagnosis of hypercholesterolemia, and use of standard lipid-lowering therapy. Exclusion criteria included any current or prior conditions affecting other bodily systems, such as familial hypercholesterolemia, severe cardiovascular and cerebrovascular diseases (e.g., myocardial infarction, stroke, and heart failure), respiratory diseases, infectious diseases, chronic kidney disease, pregnancy, and malignancies. The diagnostic criteria for hypertension were an average systolic blood pressure (SBP) ≥140 mmHg or diastolic blood pressure (DBP) ≥90 mmHg (measured three times at 5-min intervals), or 24-h SBP ≥130 mmHg or 24-h DBP ≥80 mmHg, or a history of hypertension with treatment using antihypertensive medications (12). As per the American Diabetes Association, the diagnostic criteria for diabetes included symptoms of diabetes (polyuria, polydipsia, and unexplained weight loss) and random blood glucose concentrations ≥200 mg/dL (11.1 mmol/L) from two samples, fasting glucose ≥126 mg/dL (7.0 mmol/L), or glucose concentrations ≥200 mg/dL (11.1 mmol/L) 2 h after a 75-g oral glucose tolerance test (13). According to the Chinese Guideline for Lipid Management (primary care version 2024), the diagnostic criteria for hypercholesterolemia were plasma total cholesterol (TC) ≥5.2 mmol/L or LDL-C ≥3.4 mmol/L. All participants signed written informed consent. This study adhered to the principles of the Declaration of Helsinki and was approved by the Ethics Committee of Anzhen Hospital, Capital Medical University.

### Data collection and clinical laboratory testing

Demographic characteristics, including age, sex, body mass index (BMI), and use of lipid-lowering therapy, were recorded for each participant. Fasting blood samples were collected, and plasma TC, triglyceride (TG), LDL-C, and high-density lipoprotein cholesterol (HDL-C) concentrations were measured using an automated biochemical analyzer (AU5400, Beckman Coulter, Brea, CA, United States).

### Sample preparation and liquid chromatography-mass spectrometry analysis

A 100-μL aliquot of plasma was mixed with 400 μL of ice-cold chloroform-methanol (2:1, v/v) containing stable isotope-labeled internal standard L-carnitine-d3. The mixture was vortexed for 5 min at 4°C and centrifuged at 15,000 rpm for 15 min at 4°C. After protein precipitation, the lower organic phase was collected in a clean, dry tube and evaporated to dryness. The dried residue was stored at −80°C until further analysis. Each plasma sample was characterized for lipidomic profiling. Briefly, the residue was reconstituted in 200 μL of chloroform:methanol (1:1, v/v) and diluted threefold with isopropanol:acetonitrile:water (2:1:1, v/v/v). Analysis was performed using a reverse-phase X-select CSH C18 column (2.1 × 100 mm, 2.5 μm; Waters Corp., Milford, MA, United States). The mobile phase consisted of a linear gradient system of phase A (60% acetonitrile/40% water solution containing 10 mM ammonium acetate and 0.1% formic acid) and phase B (isopropanol:acetonitrile, 9:1, v/v): 0 min 60% A, 2 min 57% A, 12 min 40% A, 12.1 min 25% A, 18 min 1% A, 19 min 1% A, and 20 min 40% A. The injection volume was 10 μL. The column temperature was maintained at 40°C at a flow rate of 0.4 mL/min. Mass spectrometry analysis was performed on a Q-Exactive HF MS (Thermo Fisher Scientific, Waltham, MA, United States) using a data-dependent acquisition model. The spray voltage was set to 3.3 kV for the positive ion mode and 2.5 kV for the negative ion mode. Each acquisition cycle included one survey scan from 150 to 1,200 m/z at a resolution of 60,000, followed by 10 MS/MS scans at a resolution of 15,000, using stepped normalized collision energy values of 15, 30, and 45. Dynamic exclusion was set to 10 s, with a sheath gas flow rate of 40 L/h and an auxiliary gas flow rate of 10 L/h. The probe heater temperature and capillary temperature were set at 300°C and 320°C, respectively.

### Mice and diets

All animal care and use procedures were approved by the Institutional Animal Care and Use Committee of Capital Medical University and were conducted in a specific-pathogen-free facility at Anzhen Hospital, Capital Medical University, Beijing, China. Eight-week-old male C57BL/6J mice and LDLR^−/−^ global knockout mice were purchased from Beijing Huafukang Biotechnology Co., Ltd. (Beijing, China). C57BL/6J mice were housed at 27°C in a controlled environment with a 12-h/12-h light/dark cycle, with free access to water and a high-cholesterol diet (HCD; Research Diets, D12108 C, 1.25% cholesterol) or an ALC-supplemented diet (300 mg/kg, Changzhou Shuiyushu’er Company, Changzhou, China) for 4 weeks (*n* = 6 mice per group). LDLR^−/−^ global knockout mice were housed in the same environment as C57BL/6J mice, with free access to water and a high-cholesterol diet (HCD; Research Diets, D12108 C, 1.25% cholesterol) or an ALC-supplemented diet (600 mg/kg, Changzhou Shuiyushu’er Company, Changzhou, China) for 8 weeks (Control group, *n* = 4; Experimental group, *n* = 10). Food intake was recorded during this period. At the end of this phase, all mice were euthanized, and body and liver weights were recorded. Tissues were dissected, cut into small pieces, and stored at −80°C.

### Biochemical assays

After fasting for 4 h, blood was collected to extract serum, and serum TC and TG concentrations were measured using kits provided by Nanjing Jiancheng Bioengineering Institute, Nanjing, China. Lipids were extracted from 20–30 mg of frozen liver samples, then 0.18–0.27 mL of anhydrous ethanol was added (volume ratio 9:1), and the tissue was homogenized at 4°C, 2,500 rpm for 10 min. The supernatant was collected, and TC and TG contents were determined using enzymatic assays from Nanjing Jiancheng Bioengineering Institute.

### Fast protein liquid chromatography

Plasma lipoprotein profiles were analyzed using fast protein liquid chromatography (FPLC). Plasma samples were collected from mice after a 12-h fasting period. Equal volumes of plasma from individual mice within each experimental group were pooled to obtain sufficient sample volumes. The pooled plasma (200 μL) was filtered through a 0.22-μm syringe filter to remove particulates. Samples were loaded onto a Superose 6 10/300 GL column (GE HealthCare) pre-equilibrated with phosphate-buffered saline (PBS) containing 0.15 M NaCl and 0.02% sodium azide. The column was connected to an ÄKTA FPLC system (GE HealthCare) and operated at a flow rate of 0.5 mL/min at room temperature. Fractions were collected every minute for 60 min. The cholesterol content in each fraction was determined using an enzymatic cholesterol assay kit (ab65390, Abcam) per the manufacturer’s instructions. The absorbance was measured at 570 nm using a microplate reader (Model 680, Bio-Rad). Lipoprotein fractions were identified based on the elution profile: very-low-density lipoprotein (VLDL) eluted in fractions 10–20, LDL in fractions 21–30, and HDL in fractions 31–50.

### En face analysis of atherosclerosis and plaque histology

For en face analysis of atherosclerotic lesions, the entire aorta was excised and stained with oil red O, and the lesion area was quantified using Fuji ImageJ software. Hearts were fixed in 4% paraformaldehyde and embedded in paraffin or OCT for histological analysis. Five-micron sections of the heart valve region were cut and stained with hematoxylin and eosin (H&E), and the atherosclerotic lesions were analyzed using Fuji ImageJ software across six consecutive sections from four to six littermates per group.

### Liver histology

Liver samples were fixed in 4% paraformaldehyde for ≥24 h, rapidly frozen, and embedded in OCT compound. Eight-micron frozen sections were prepared and stained with H&E and oil red O.

### Cell culture

Huh-7 cells were obtained from the National Collection of Authenticated Cell Cultures and cultured in Dulbecco’s modified Eagle medium (DMEM; Gibco, United States) supplemented with 10% fetal bovine serum, 2 mM L-glutamine, 100 μg/mL penicillin, and 100 μg/mL streptomycin in a 37°C incubator with 5% CO_2_.

### Western blot analysis

Protein samples were extracted from mouse liver tissue lysates and Huh-7 cells. Twenty milligrams of liver tissue samples were lysed in RIPA buffer (Solarbio, Beijing, China) using ultrasonic disruption. After centrifugation at 12,000 rpm for 20 min, the supernatant was collected for protein quantification via western blot analysis. Proteins were separated by sodium dodecyl sulfate-polyacrylamide gel electrophoresis and transferred to polyvinylidene fluoride membranes. Membranes were blocked with 5% non-fat milk and incubated sequentially with the appropriate primary and secondary antibodies. Supplementary Table S1 lists the antibodies used.

### Real-time quantitative PCR

Real-time PCR was performed to quantify specific mRNA. RNA was isolated from liver and cell samples using TRIzol reagent (Life Technologies, Thermo Fisher Scientific, Waltham, MA, United States) and converted to cDNA using a reverse transcription kit from Thermo Fisher Scientific. RT-PCR was conducted using the SYBR Green method on a real-time PCR system. The relative abundance of each mRNA species was evaluated using the $2^{-\Delta \Delta C_{T}}$ method. Supplementary Figure S1 lists the primers used.

### Immunofluorescence staining

Cells were seeded in 6-well chamber slides and incubated in complete DMEM for 24 h, then treated for another 24 h. Cells were fixed with 4% paraformaldehyde for 30 min and washed with non-permeabilizing or permeabilizing PBS containing 0.025% Triton-X. Cells were then blocked with 1% bovine serum albumin for 30 min and stained with anti-GFP antibody in PBS containing 1% bovine serum albumin for 1 h. After washing, cells were incubated with FITC-labeled goat anti-rabbit IgG (H + L) (Beyotime, Shanghai, China) and DAPI for nuclear staining. Cells were subsequently mounted with PermaFluor and visualized using an inverted fluorescence microscope (Nikon Eclipse Ti) at 20× or 40× magnification.

### Dil-LDL uptake assay

Huh-7 cells were seeded in black transparent-bottom 96-well plates for 24 h and treated with experimental reagents for an additional 24 h. During the last 5 h of treatment (19–24 h), cells were exposed to 3,3’-dioctadecylindocarbocyanine-labeled low-density lipoprotein (Dil-LDL, 10 μg/mL), then washed twice with pre-warmed (37°C) HBSS containing 20 mM HEPES before analysis. The intracellular fluorescence intensity of the Dil was quantified using the PerkinElmer Operetta CLS imaging system.

### Statistical analysis

Statistical analyses were performed using SPSS (version 26; IBM Corp., Armonk, NY, USA). Measurement data conforming to a normal distribution were expressed as mean ± standard deviation, while data not conforming to a normal distribution were expressed as median (interquartile range). For data conforming to a normal distribution, comparisons between two groups were conducted using the independent samples *t*-test, and comparisons among multiple groups were performed using one-way analysis of variance (ANOVA). For data not conforming to a normal distribution, comparisons between two groups were conducted using the Mann–Whitney *U* test, and comparisons among multiple groups were performed using the Kruskal–Wallis (*H*) test. Categorical data were described using frequencies and percentages, and differences between groups were analyzed using the chi-square test or Fisher’s exact test. A two-sided *p*-value <0.05 was considered statistically significant.

## Results

### Demographic and clinical characteristics of all participants

In total, 494 participants (mean age: 43 years, 71.05% male) were enrolled in this study (Table 1). The mean BMI of the participants was 28.0 (25.6, 31.0) kg/m^2^. The prevalence of hypertension was low (16.80%), as were the historical incidences of cardiovascular disease (9.51%) and prior lipid-lowering therapy (14.37%). The lipid profiles were favorable, with levels of LDL-C at 3.03 mmol/L, HDL-C at 1.15 mmol/L, TC at 4.94 mmol/L, and TGs at 1.67 mmol/L. However, the prevalence of diabetes mellitus (fasting blood glucose concentration of ≥126 mg/dL or 7.0 mmol/L) was relatively high (27.53%). Given these baseline characteristics, we investigated potential metabolic factors influencing lipid levels in this population.

**Table 1:** Baseline characteristics of all subjects in the discovery cohort.

Baseline characteristics	All subjects (*N* = 494)^a^	Tertile 1 (*n* = 165)^a^	Tertile 2 (*n* = 165)^a^	Tertile 3 (*n* = 164)^a^	*p*-value^b^
Ages (years)	43 (36, 54)	41 (35, 52)	45 (37, 55)	45 (35, 55)	0.18
Male (%)	71.05	71.52	72.73	68.90	0.74
BMI (kg/m^2^)	28.0 (25.6, 31.0)	28.4 (26.6, 31.2)	27.7 (25.4, 30.4)	28.0 (25.5, 31.5)	0.22
Diabetes mellitus (%)	27.53	23.03	28.48	31.10	0.25
Hypertension (%)	16.80	16.36	14.55	19.51	0.48
History of CVD (%)	9.51	9.09	11.52	7.93	0.53
History of LLT (%)	14.37	12.12	18.79	12.20	0.14
LDL cholesterol (mg/dL)	3.03 (2.49, 3.66)	3.75 (3.22, 4.12)	2.97 (2.50, 3.43)	2.52 (2.11, 2.99)	<0.001
HDL cholesterol (mg/dL)	1.15 (0.98, 1.33)	1.15 (1.00, 1.34)	1.10 (0.95, 1.29)	1.16 (1.01, 1.33)	0.11
Total cholesterol (mg/dL)	4.94 (1.00)	5.60 (0.87)	4.78 (0.89)	4.43 (0.85)	<0.001
Triglycerides (mg/dL)	1.67 (1.20, 2.49)	1.67 (1.23, 2.52)	1.71 (1.29, 2.63)	1.64 (1.08, 2.23)	0.169

BMI, body mass index; CVD, cardiovascular disease; HDL, high density lipoprotein; LDL, low density lipoprotein; LLT, lipid-lowering therapy.

a Median (IQR); mean (SD); *n* (%).

b Kruskal–Wallis rank sum test; one-way ANOVA; Pearson’s chi-squared test.

### Serum ALC was negatively correlated with LDL-C in hypercholesterolemic individuals

**Figure 1:**
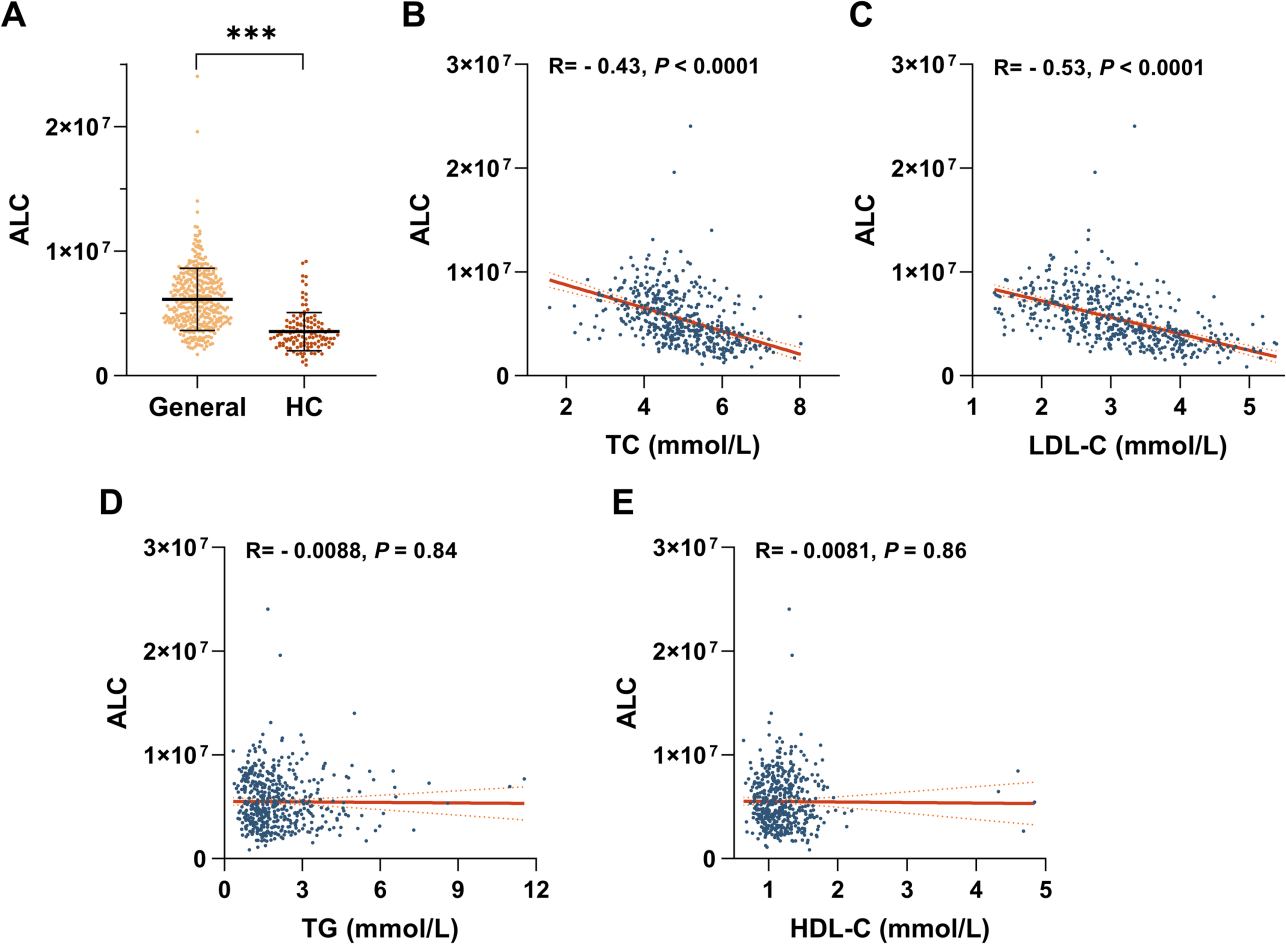
Negative correlation between ALC and LDL-C in hypercholesterolemic patients. **(A)** Plasma ALC levels in hypercholesterolemic patients compared with the general population. Regression analyses of ALC versus plasma TC concentrations **(B)**, plasma LDL-C concentrations **(C)**, plasma TG concentrations **(D)**, and plasma HDL-C concentrations **(E)**. Comparisons between two groups were made using Student’s *t*-test, ^*^p < 0.05; ^**^p < 0.01, and ^***^p < 0.001.

Metabolomic qualitative and quantitative assessments of the patients’ plasma and clinical characteristics revealed that plasma ALC was significantly downregulated. Individuals with hypercholesterolemia exhibited approximately 42% lower plasma ALC levels than those of the general population (Figure 1A). ALC was significantly negatively correlated with TC (*r* = −0.43, *p* < 0.0001; Figure 1B) and LDL-C (*r* = −0.53, *p* < 0.0001; Figure 1C) but not significantly correlated with TGs (*r* = −0.0088, *p* < 0.0001; Figure 1D) or HDL-C (*r* = −0.0081, *p* < 0.0001; Figure 1E). These findings suggest a potential role of ALC in modulating cholesterol levels. To further explore this relationship, we conducted animal studies.

### ALC reduced plasma TC and LDL-C levels in C57BL/6J mice fed a high-cholesterol diet

**Figure 2:**
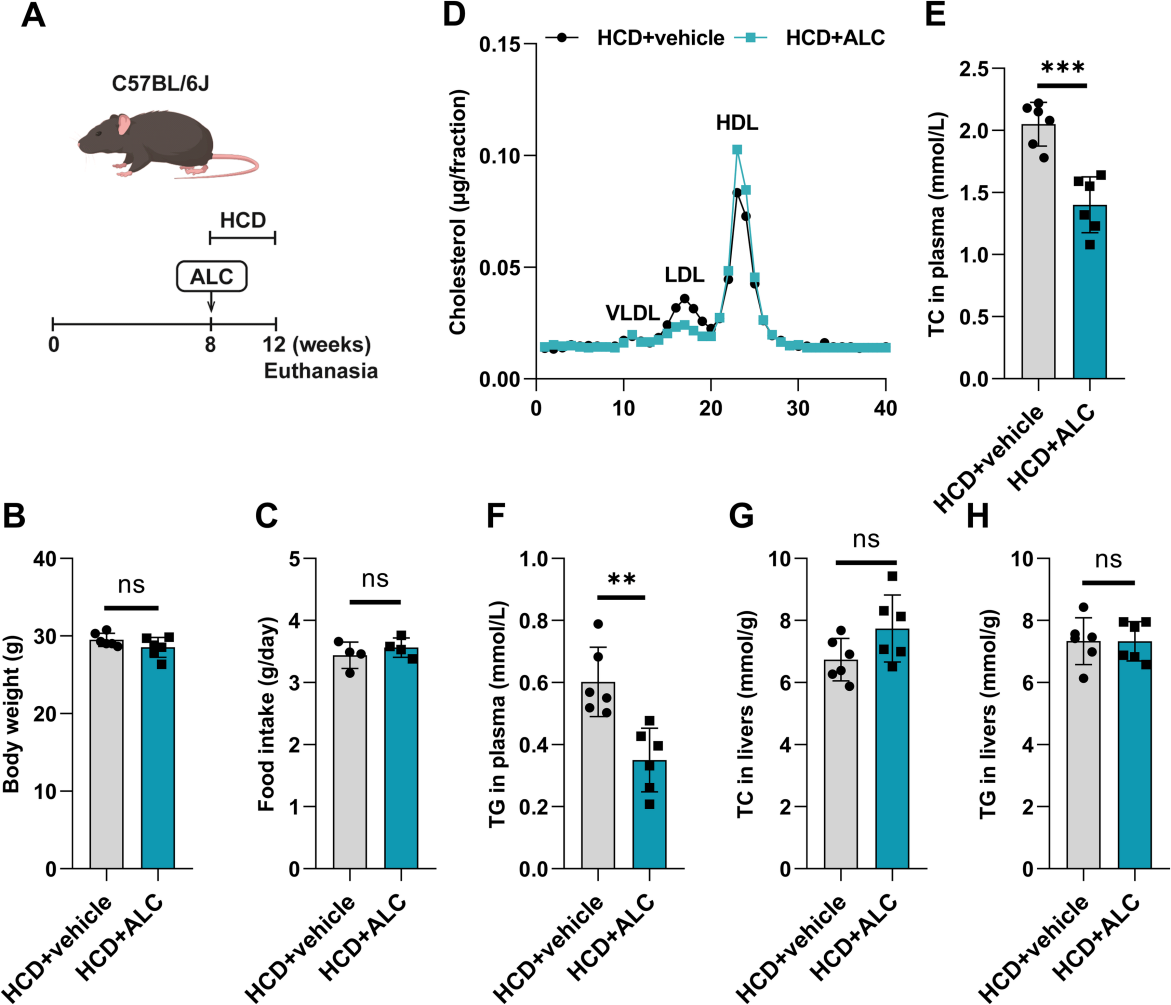
ALC reduced plasma cholesterol levels in mice. **(A)** Eight-week-old male C57BL/6J mice (*n* = 6 per group) were fed a high-cholesterol diet (HCD group) or a high-cholesterol diet supplemented with 300 mg/kg ALC (HCD + ALC group) for 4 weeks; **(B)** comparison of mouse body weights; **(C)** comparison of food intake among mice; **(D)** quantification of VLDL, LDL, and HDL contents in the cholesterol via FPLC. Enzymatic assays for plasma TC levels in mice **(E)**, plasma TG levels in mice **(F)**, liver TC levels in mice **(G)**, and liver TG levels in mice **(H)**. Data are presented as means ± SEM. Comparisons between two groups were made using Student’s *t*-test, ^**^p < 0.01 and ^***^p < 0.001.

Building upon the clinical observations, among the differential plasma metabolites of patients with hyperlipidemia, ALC was significantly downregulated and negatively correlated with plasma LDL-C. To investigate whether ALC could reduce plasma lipid levels in hyperlipidemic mice, we administered ALC to C57BL/6J mice to observe its effect on plasma lipids. ALC was incorporated into D12108 feed containing 1.25% cholesterol to produce an HCD with an ALC concentration of 300 mg/kg. This diet was provided to 8-week-old male C57BL/6J mice (the HCD + ALC group) for 4 weeks (Figure 2A). A control group received the HCD without ALC supplementation. Compared with the HCD group, the HCD + ALC group showed no significant changes in body weight or food intake (Figures 2B,C), indicating that ALC did not affect overall health status. FPLC analysis revealed that plasma LDL-C in the HCD + ALC group exhibited a decreasing trend, whereas HDL-C showed an increasing trend (Figure 2D). TC and TG levels in the mouse plasma and livers were measured using enzymatic assays. Compared with the HCD group, the HCD + ALC group had significantly reduced plasma TC and TG levels (Figures 2E,F). However, hepatic TC and TG levels did not significantly differ (Figures 2G,H). These results indicate that ALC effectively reduced plasma lipid levels without altering hepatic lipid storage.

### ALC regulated hepatic mRNA and protein levels of cholesterol metabolism-related genes in mice

**Figure 3:**
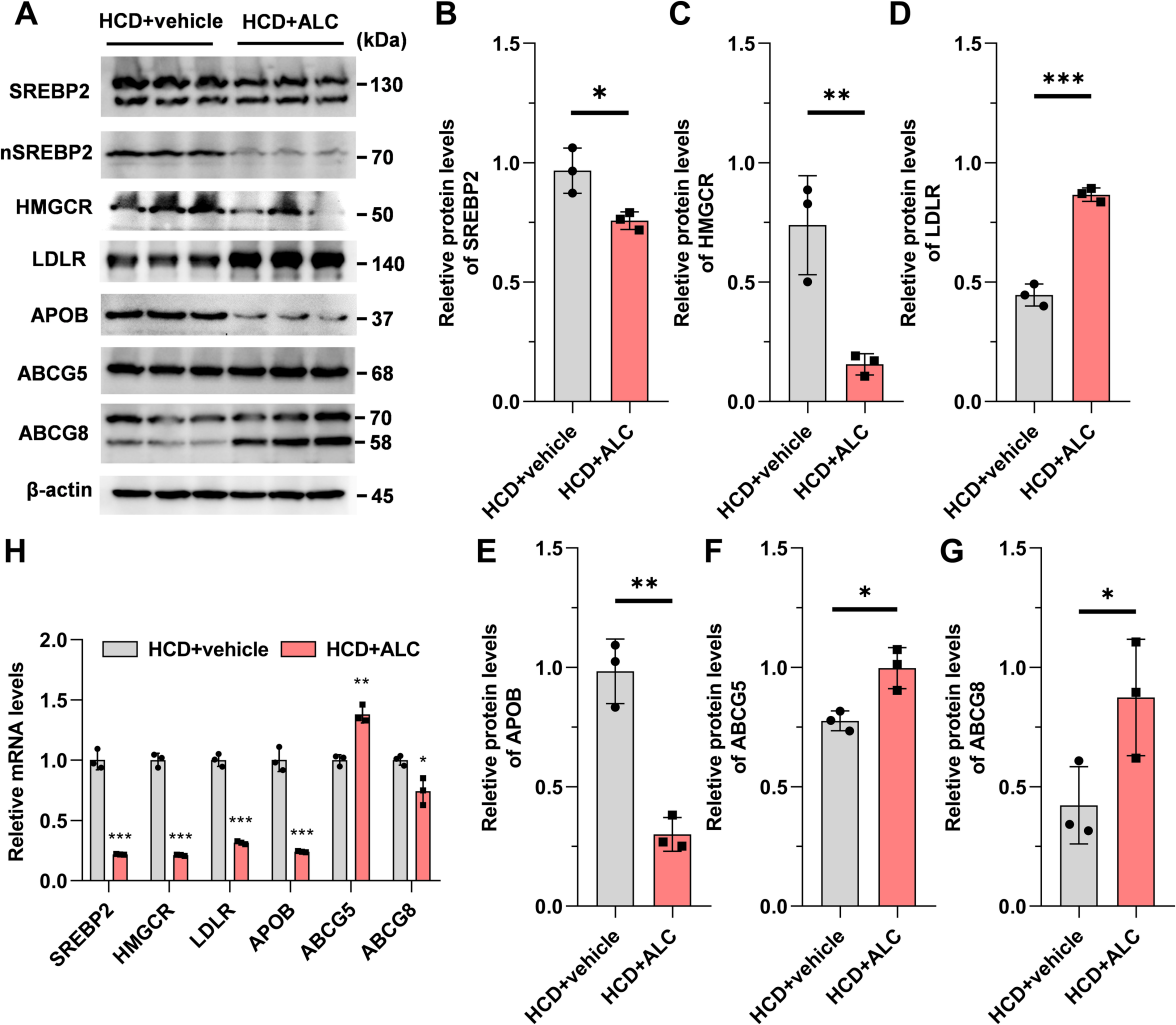
Effects of ALC on expressions of genes and proteins involved in cholesterol metabolism pathways. Eight-week-old male C57BL/6J mice (*n* = 6 per group) were fed a high-cholesterol diet (HCD group) or a high-cholesterol diet supplemented with 300 mg/kg ALC (HCD + ALC group) for 4 weeks. Western blot and qRT-PCR were used to measure liver protein and mRNA expression: **(A)** representative images from western blot; **(B)** SREBP2; **(C)** HMGCR; **(D)** LDLR; **(E)** APOB; **(F)** ABCG5; **(G)** ABCG8; and **(H)** mRNA expressions of these genes. Data are presented as means ± SEM. Comparisons between two groups were made using Student’s *t*-test, ^*^p < 0.05, ^**^p < 0.01, and ^***^p < 0.001.

To clarify the mechanism by which ALC affects plasma lipid levels in mice, we analyzed hepatic gene and protein expression related to cholesterol metabolism. Hepatic protein and mRNA levels were assessed using western blot analysis (Figure 3A) and qRT-PCR (Figure 3H), respectively. Compared with the HCD group, the HCD + ALC group exhibited significantly decreased hepatic protein levels of SREBP2 (Figure 3B), 3-hydroxy-3-methyl-glutaryl-CoA reductase (HMGCR; Figure 3C), and APOB (Figure 3E) and significantly increased levels of ABCG5 (Figure 3F), ABCG8 (Figure 3G), and low-density lipoprotein receptor (LDLR; Figure 3D). Furthermore, compared with the HCD group, the HCD + ALC group showed significantly decreased *Srebp2*, *Hmgcr*, *Ldlr*, *Apob*, and *Abcg8* mRNA levels and a significantly increased *Abcg5* mRNA level (Figure 3H). These alterations suggest that ALC modulates key regulators of cholesterol synthesis and transport at the transcriptional and translational levels.

### ALC lowered plasma TC levels in LDLR^−/−^ mice

**Figure 4:**
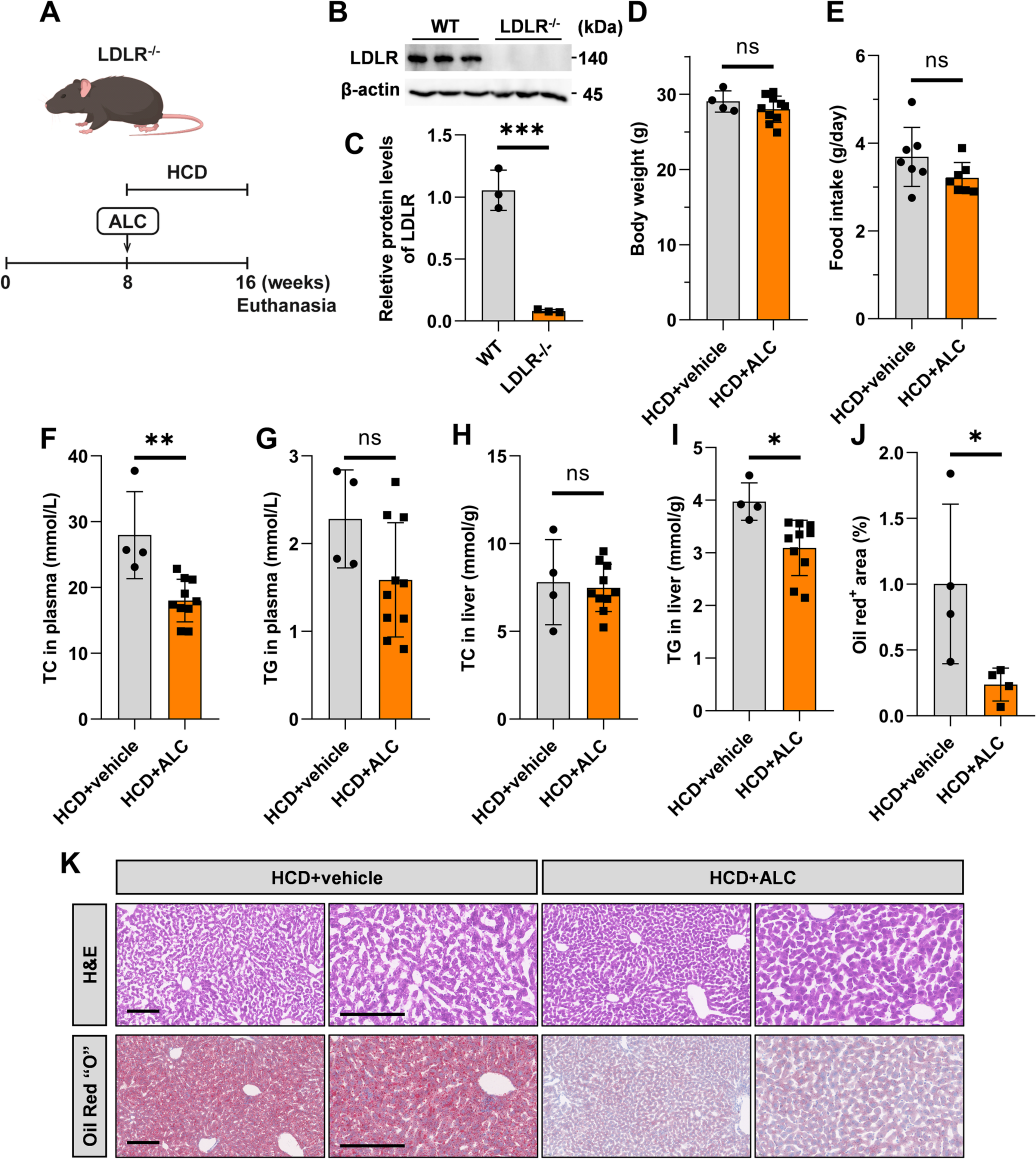
ALC reduced plasma TC levels in LDLR^−/−^ mice. **(A)** Eight-week-old male LDLR^−/−^ mice were fed a high-cholesterol diet (HCD group, *n* = 4) or a high-cholesterol diet supplemented with 300 mg/kg ALC (HCD + ALC group, *n* = 10) for 8 weeks; **(B,C)** western blot analysis of LDLR protein levels in mouse livers; **(D)** comparison of mouse body weights; **(E)** comparison of food intake among mice. Enzymatic assays for plasma TC levels in mice **(F)**, plasma TG levels in mice **(G)**, liver TC levels in mice **(H)**, and liver TG levels in mice **(I)**. **(J)** Analysis of oil red O-stained areas in the liver; **(K)** H&E and oil red O staining of the liver. Data are presented as means ± SEM. Comparisons between two groups were made using Student’s *t*-test, ^*^p < 0.05, ^**^p < 0.01, and ^***^p < 0.001.

To evaluate ALC’s efficacy in a hypercholesterolemic model prone to atherosclerosis, HCD-fed LDLR^−/−^ mice provide a well-established animal model of hypercholesterolemia, which is an independent risk factor for atherosclerosis. To establish a mouse model of hypercholesterolemia-induced atherosclerosis, we fed 8-week-old male LDLR^−/−^ mice D12108 feed containing 1.25% cholesterol for 8 weeks. From week 8 onward, the mice were divided into two groups: one group continued on the established HCD; the other received the same diet supplemented with 300 mg/kg ALC for an additional 8 weeks (HCD + ALC group; Figure 4A). This allowed us to assess the impact of ALC on established hyperlipidemia. Western blot analysis revealed the hepatic LDLR expression levels. Expectedly, LDLR^−/−^ mice exhibited significantly reduced hepatic LDLR levels compared with those of the wild-type mice (Figures 4B,C). Body weight and food intake did not significantly differ between the HCD and HCD + ALC groups (Figures 4D,E). Enzymatic assays revealed the plasma and hepatic TC and TG levels. The HCD + ALC group showed significantly decreased plasma TC levels compared with those of the HCD group, whereas plasma TG levels remained unchanged (Figures 4F,G). Hepatic TC levels did not significantly differ between the two groups (Figure 4H); however, hepatic TG levels were significantly reduced in the HCD + ALC group (Figure 4I). Liver sections were stained with H&E and oil red O (Figure 4K). Compared with the HCD group, the HCD + ALC group exhibited reduced hepatocyte vacuolation and significantly decreased oil red O-positive staining in the liver sections (Figure 4J). These findings demonstrate that ALC effectively lowers plasma cholesterol and reduces hepatic lipid accumulation even in the absence of LDL receptors.

### ALC attenuated atherosclerosis in LDLR^−/−^ mice

**Figure 5:**
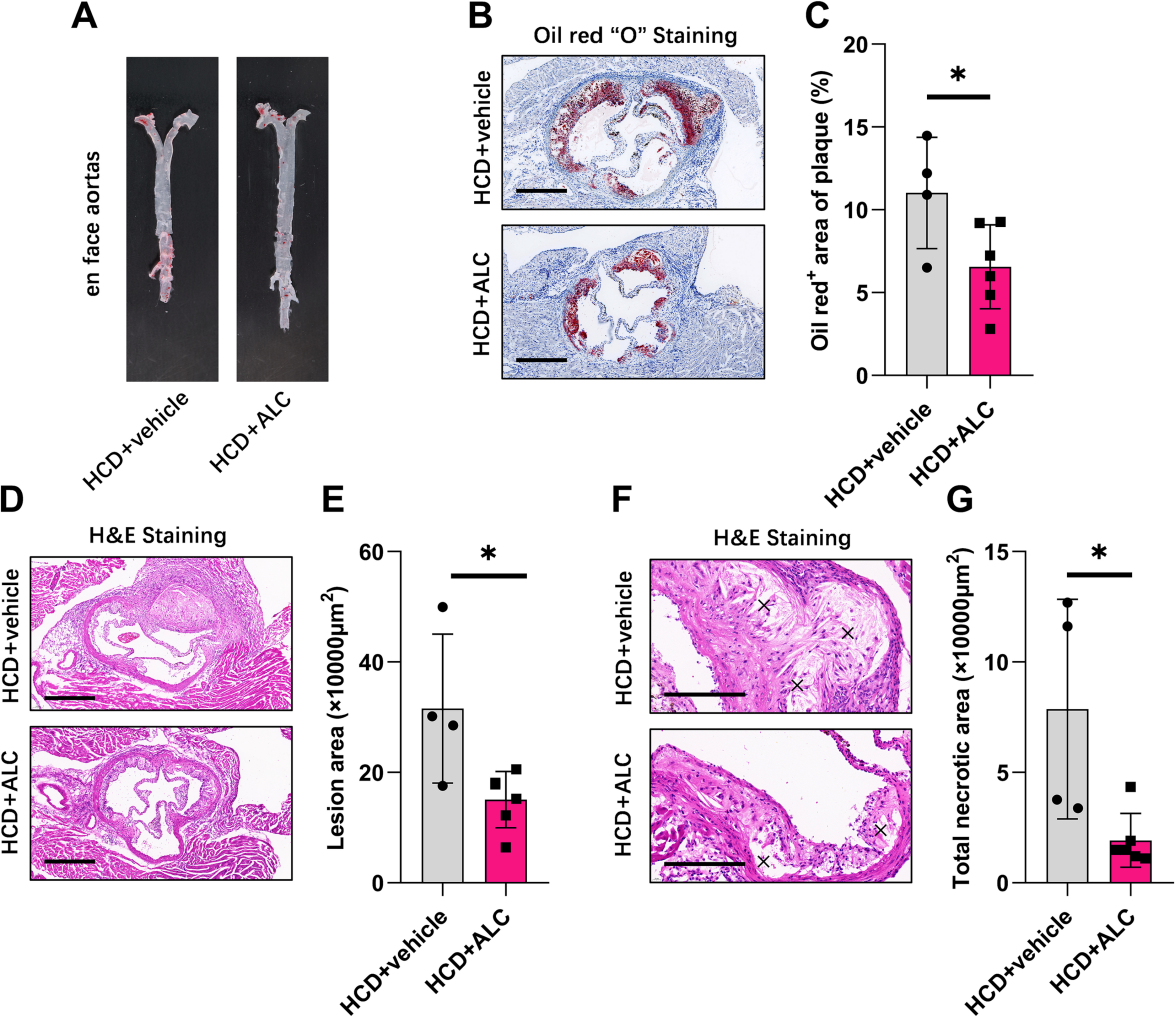
ALC mitigated atherosclerotic progression in LDLR^−/−^ mice. Eight-week-old male LDLR^−/−^ mice were fed a high-cholesterol diet (HCD group, *n* = 4) or a high-cholesterol diet supplemented with 300 mg/kg ALC (HCD + ALC group, *n* = 10) for 8 weeks. **(A)** Representative images of oil red O staining in the aortas of mice; **(B)** representative images of cross-sectional oil red O staining at the aortic root, scale bar = 500 μm; **(C)** quantification of the percentage of oil red O-positive areas indicating lesion areas; **(D–G)** frozen sections of the aortic root stained with H&E; **(D)** representative image, scale bar = 500 μm; **(E)** quantification of lesion area; **(F)** representative image, scale bar = 200 μm; **(G)** quantification of necrotic areas, denoted by an “×”. Comparisons between two groups were made using Student’s *t*-test, ^*^p < 0.05 and ^**^p < 0.01.

To investigate whether ALC influences atherosclerotic progression, we analyzed aortic root sections from mice using H&E and oil red O staining. Oil red O en face staining of the entire aorta demonstrated a reduced atherosclerotic plaque area in the HCD + ALC group compared with that of the HCD group (Figure 5A). Similarly, oil red O staining of aortic root cross-sections showed a significantly decreased oil red O-positive staining area in the HCD + ALC group compared with that of the HCD group (Figures 5B,C). H&E staining revealed that the atherosclerotic lesion areas in the aortic root cross-sections were significantly reduced in the HCD + ALC group compared with the HCD group (Figures 5D,E). Additionally, the total necrotic area was significantly decreased in the HCD + ALC group (Figures 5F,G). These results suggest that ALC supplementation mitigates atherosclerosis development in hypercholesterolemic conditions.

### ALC reduced cholesterol synthesis by decreasing SREBP2 levels in Huh-7 hepatocytes

**Figure 6:**
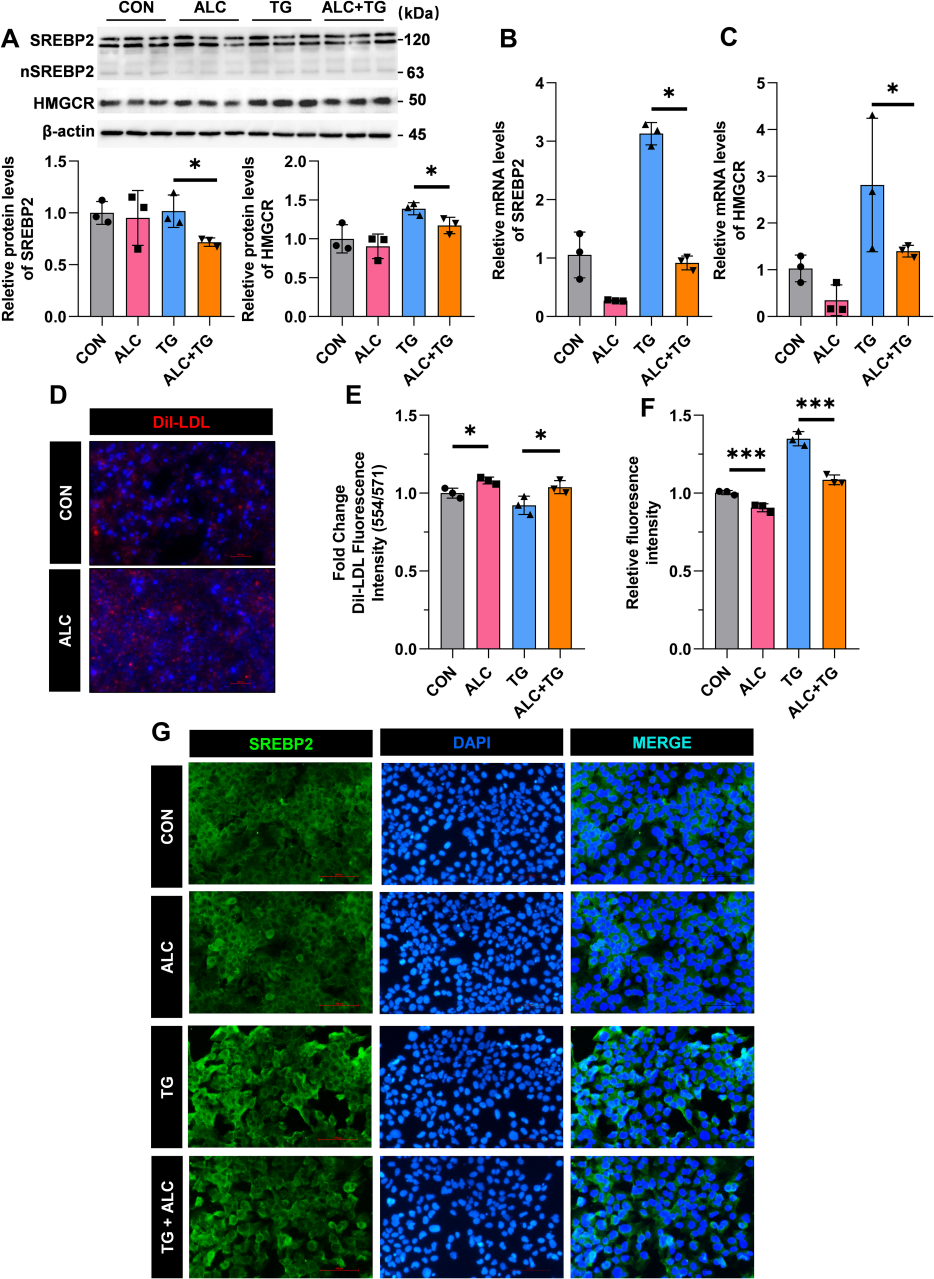
ALC inhibited SREBP2 activation in hepatocytes. Huh-7 cells were treated with the SREBP2 agonist, TOFA (TG; 100 nM), in the presence or absence of ALC (100 μM) for 24 h. **(A–C)** Western blot and qRT-PCR analysis of SREBP2 and HMGCR protein **(A,B)** and mRNA **(C)** expressions in Huh-7 and HepG2 cells; **(D)** representative image of Dil-LDL staining; **(E)** changes in Dil-LDL fluorescence intensity; **(F,G)** immunofluorescence detection of SREBP2 expression; **(F)** quantitative analysis of fluorescence intensity; **(G)** representative immunofluorescence images. One-way ANOVA was used for multiple-group comparisons, ^*^p < 0.05 and ^***^p < 0.001.

To elucidate the cellular mechanisms underlying ALC’s cholesterol-lowering effects, ALC reduced the plasma TC in mice and decreased the hepatic SREBP2 and HMGCR levels. Therefore, we hypothesized that ALC may lower plasma TC levels by reducing cholesterol synthesis in hepatocytes. Thapsigargin is a common inducer of endoplasmic reticulum (ER) stress. Previous studies have shown that thapsigargin-induced ER stress increases SREBP2 levels in Huh-7 hepatocytes (14). We treated Huh-7 cells for 24 h with medium containing 100 nM TG, with or without addition of 100 mM ALC. Cells were divided into the control (CON), ALC, TG, and ALC + TG groups. Western blotting and qRT-PCR were performed to assess the protein and mRNA SREBP2 and HMGCR levels. Compared with the TG group, the ALC + TG group exhibited significantly decreased protein and mRNA SREBP2 and HMGCR levels (Figures 6A–C). Cholesterol uptake was assessed using Dil-LDL; the Dil-LDL fluorescence intensity was significantly increased in the ALC group compared with the CON group and significantly decreased in the ALC + TG group compared with the TG group (Figures 6D,E). Immunofluorescence analysis of SREBP2 expression in Huh-7 cells showed that compared with the CON group, the ALC group had significantly reduced SREBP2 levels, and similarly, compared with the TG group, the ALC + TG group had significantly reduced SREBP2 levels (Figures 6F,G). These *in vitro* findings support the notion that ALC decreases cholesterol synthesis by downregulating SREBP2, thereby contributing to its lipid-lowering effects observed *in vivo*.

## Discussion

Our results revealed that plasma ALC levels were negatively correlated with cholesterol levels in individuals with hypercholesterolemia. To explore the effect of ALC on cholesterol, we administered ALC to both wild-type and LDLR^−/−^ mice. ALC supplementation significantly reduced plasma TC and LDL-C levels in HCD-fed mice and attenuated atherosclerotic progression in LDLR^−/−^ mice. In the livers of ALC-treated mice, cholesterol metabolism-related genes exhibited changes, with significant decreases in SREBP2 and HMGCR, key components of the cholesterol synthesis pathway. In hepatocytes, SREBP2 activation increased HMGCR transcription, but ALC reverses this trend. These findings suggest that ALC may reduce *de novo* cholesterol synthesis by inhibiting SREBP2, leading to decreased HMGCR levels and subsequently lowering cholesterol. Therefore, ALC may be a potential cholesterol-lowering agent.

ALC, approved by the U.S. Food and Drug Administration, is now widely used as a dietary supplement (15, 16). In China, ALC is undergoing phase III clinical trials as a medication to alleviate paresthesia caused by diabetic peripheral neuropathy (17). Diabetes mellitus is an important independent risk factor for ASCVD, a common complication and a leading cause of mortality in diabetic patients. The prevalence of dyslipidemia is significantly higher in diabetic patients, especially those with type 2 diabetes, than in non-diabetic individuals (18, 19). Adult patients with diabetes should begin pharmacotherapy along with lifestyle interventions to achieve lipid-lowering targets early and reduce ASCVD events. Although the current use of statins, cholesterol absorption inhibitors, and PCSK9 inhibitors has greatly increased the rate of LDL-C goal attainment, safe and effective lipid-lowering drugs are still needed.

The liver is the primary site of cholesterol synthesis (20). SREBP2 is a major participant in the cholesterol biosynthesis pathway, functioning as a key transcriptional regulator of cholesterol synthesis (21, 22). SREBP2 is synthesized in the ER, then translocates to the Golgi apparatus, where it is processed into the active nuclear form, nSREBP2 (23–25). The processed nSREBP2 enters the nucleus and binds to sterol regulatory element sequences in target gene promoters, including HMGCR, upregulating their transcription (26). Previous studies have found that mechanistic target of rapamycin complex 1 (mTORC1) increases nSREBP2 levels through phosphorylation (27). Other studies have shown that the lipogenic transcription factor carbohydrate-responsive element-binding protein (ChREBP) promotes ubiquitination and proteasomal degradation of nSREBP2 through an unexplored mechanism (28). HMGCR is a glycoprotein localized in the ER and is the rate-limiting enzyme in cholesterol biosynthesis. It is highly regulated at transcriptional, translational, and post-translational levels (29). Here, we report that ALC reduced SREBP2 and HMGCR expression in mouse livers and human hepatoma cells and increased the expression of cholesterol metabolism-related genes, such as *Ldlr*, in the livers of ALC-fed mice. Our findings suggest that ALC may lower cholesterol by reducing SREBP2 and nSREBP2 levels, consequently decreasing HMGCR and reducing *de novo* cholesterol synthesis. Therefore, ALC may be a potential cholesterol-lowering agent.

ALC belongs to the acylcarnitine family and is an endogenous molecule that plays an important role in lipid energy metabolism within the mitochondria (30, 31). A previous clinical study reported decreased plasma ALC levels in 174 individuals with early-onset CVD (32). Our previously published work revealed that plasma ALC levels were lower in individuals with familial hypercholesterolemia (33). The current study revealed that plasma ALC levels were approximately 42% lower in individuals with hypercholesterolemia compared to those without hypercholesterolemia. Further analysis revealed that plasma ALC levels were negatively correlated with TC (*r* = −0.43) and LDL-C (*r* = −0.53), suggesting a potential association between ALC and plasma cholesterol levels. Because cholesterol is an independent risk factor for ASCVD, and ALC is associated with cholesterol reduction, decreased ALC levels may contribute to ASCVD risk.

Our study has several limitations. First, despite significant TC and LDL-C reduction in LDLR^−/−^ mice treated with ALC, their plasma TG levels remained higher than those of wild-type mice. Longer-term ALC administration was not feasible due to resource constraints. Second, our reliance on traditional methodologies may limit mechanistic insights; future studies incorporating high-throughput approaches (e.g., RNA sequencing) are warranted. Third, the correlational design and limited sample size of our clinical study preclude definitive conclusions; larger, longitudinal studies are needed to validate the observed ALC-cholesterol association and investigate causality regarding ASCVD risk.

In conclusion, we found that ALC significantly reduced SREBP2 and HMGCR expression, leading to decreased *de novo* cholesterol synthesis, lower serum cholesterol levels in HCD-fed mice, and attenuated atherosclerotic progression. Despite the limitations mentioned, our findings provide novel insights into the potential use of ALC for lowering cholesterol and preventing atherosclerosis. Future studies should explore the long-term effects of ALC supplementation, use high-throughput techniques to uncover detailed mechanisms, and conduct large-scale clinical trials to confirm its therapeutic potential.

## Data Availability

The raw data supporting the conclusions of this article will be made available by the authors, without undue reservation.
