# Supplementary material for: Acetyl-L-carnitine ameliorates atherosclerosis in LDLR−/− mice by modulating cholesterol metabolism through SREBP2-dependent cholesterol biosynthesis
Source: Front Nutr. 2024 Dec 16;11:1509577. doi: 10.3389/fnut.2024.1509577 (PMC11684389; doi:10.3389/fnut.2024.1509577)
Supplement: Supplementary file 3 [file Image_1.pdf]

Supplementary Figure

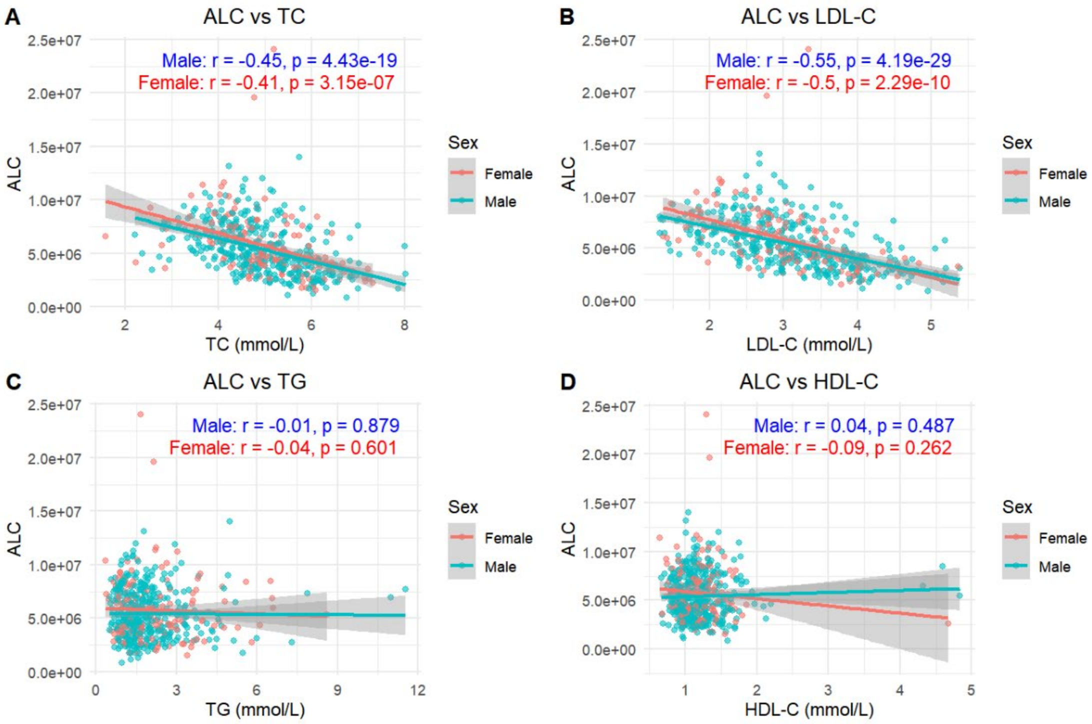

Figure Legend: ALC vs lipid parameters stratified by sex.
A: ALC vs TC, B: ALC vs LDL-C, C: ALC vs TG, D: ALC vs HDL

The sex-stratified analysis revealed significant negative correlations between ALC and TC in both males ($r = -0.45$, $p < 0.001$) and females ($r = -0.41$, $p < 0.001$). Similarly, ALC was negatively correlated with LDL-C in both males ($r = -0.55$, $p < 0.001$) and females ($r = -0.50$, $p < 0.001$). However, no significant correlations were observed between ALC and TG (males: $r = -0.01$, $p = 0.879$; females: $r = -0.04$, $p = 0.601$) or HDL-C (males: $r = 0.04$, $p = 0.487$; females: $r = -0.09$, $p = 0.262$) in either group. These results suggest that the relationships between ALC and TC/LDL-C are consistent across sexes, while TG and HDL-C show no significant associations with ALC.
